# DIFFERENCES IN SELF-CARE AND QUALITY OF LIFE BETWEEN SPOUSES AND ADULT CHILDREN IN PATIENTS WITH PARKINSON’S DISEASE

**DOI:** 10.1093/geroni/igae098.2826

**Published:** 2024-12-31

**Authors:** Juhee Lee, Eunyoung Kim, Hyeran Park, Kiyeon Kim

**Affiliations:** Yonsei University, Seoul, Republic of Korea; Yonsei University College of Nursing, Seoul, Republic of Korea; Yonsei University College of Nursing, Seoul, Republic of Korea; Yonsei University College of Nursing, Seoul, Republic of Korea

## Abstract

Self-care of patients with chronic illness involves various caregiver types like spouses, adult children, and other relatives or friends. Several studies have reported differences in self-care and quality of life (QOL) according to caregiver type in patients with chronic illness. Nevertheless, there are few studies comparing the difference by caregiver type in patients with Parkinson’s disease (PD). This is a cross-sectional study to explore differences in self-care and QOL between spouse and adult child caregivers of patients with PD. Participants were recruited from two neurology outpatient departments of the tertiary hospitals in Korea. They reported self-care and QOL using a structured questionnaire. A total of 111 paired of PD patients and caregivers including 83 of spouse and 28 of adult child were analyzed using SPSS 26.0. Spouse caregivers reported higher in domains for self-care of efficacy and monitoring in both patients (p=0.014 to 0.054) and caregivers (p=0.026 to 0.002) compared with adult child caregivers. Patients with spouse caregivers showed better QOL of parkinson symptoms(p=0.008), and emotional function(p=0.003) with the exception of social functioning (p=0.31). Social functioning contained the questionnaire like hobby, vacation, or sexuality. This study finding showed that patients whose caregivers are spouses had better in both self-care and QOL domain than patients whose caregivers are adult children. These differences between spouse caregivers and adult child caregivers can suggest the needs of tailored care strategy for patients with PD according to caregiver type.

